# Performance-based financing in the context of the complex remuneration of health workers: findings from a mixed-method study in rural Sierra Leone

**DOI:** 10.1186/s12913-016-1546-8

**Published:** 2016-07-19

**Authors:** Maria Paola Bertone, Mylene Lagarde, Sophie Witter

**Affiliations:** Department of Global Health and Development, London School of Hygiene and Tropical Medicine, London, UK; ReBUILD Consortium Affiliate, London School of Hygiene and Tropical Medicine, London, UK; ReBUILD Consortium, IIHD, Queen Margaret University, Edinburgh, UK

**Keywords:** Performance-based financing, Remuneration, Financial incentives, Health workers, Sierra Leone

## Abstract

**Background:**

There is growing interest on the impact of performance-based financing (PBF) on health workers’ motivation and performance. However, the literature so far tends to look at PBF payments in isolation, without reference to the overall remuneration of health workers. Taking the case of Sierra Leone, where PBF was introduced in 2011, this study investigates the absolute and relative contribution of PBF to health workers’ income and explores their views on PBF bonuses, in comparison to and interaction with other incomes.

**Methods:**

The study is based on a mixed-methods research consisting in a survey and an 8-week longitudinal logbook collecting data on the incomes of primary health workers (*n* = 266) and 39 in-depth interviews with a subsample of the same workers, carried out in three districts of Sierra Leone (Bo, Kenema and Moyamba).

**Results:**

Our results show that in this setting PBF contributes about 10 % of the total income of health workers. Despite this relatively low contribution, their views on the bonuses are positive, especially compared to the negative views on salary. We find that this is because PBF is seen as a complement, with less sense of entitlement compared to the official salary. Moreover, PBF has a specific role within the income utilization strategies enacted by health workers, as it provides extra money which can be used for emergencies or reinvested in income generating activities. However, implementation issues with the PBF scheme, such as delays in payment and difficulties in access, cause a series of problems that limit the motivational effects of the incentives. Overall, staff still favor salary increases over increases in PBF.

**Conclusions:**

The study confirms that the remuneration of health workers is complex and interrelated so that the different financial incentives cannot be examined independently from one. It also shows that the implementation of PBF schemes has an impact on the way it does or does not motivate health workers, and must be thoroughly researched in order to assess the impact of PBF.

**Electronic supplementary material:**

The online version of this article (doi:10.1186/s12913-016-1546-8) contains supplementary material, which is available to authorized users.

## Background

Performance-based financing (PBF) schemes are implemented in a growing number of low and middle-income countries and in particular in sub-Saharan Africa. At the moment, there are about 34 countries in Africa where at least one pilot or regional scheme is in place [1]. In a few countries, notably Burundi, Rwanda and Sierra Leone, the setting of this research, PBF has been introduced at national level. In a nutshell, PBF schemes entail the payment of a financial bonus to healthcare providers based on their performance, measured by the quantity of services provided (or the achievement of a coverage target), out of a list of pre-identified indicators, usually adjusted by a measure of structural quality. The performance bonus is normally used to cover facility running costs and individual staff incentives. PBF is envisaged to improve the quantity and quality of services provided by increasing the motivation of health workers and their responsiveness to patients’ needs. It is also expected to have positive systemic effects through the reorganization and clarification of roles and responsibilities between actors and increased autonomy of providers, transparency and accountability [2, 3].

Research on the impact of PBF schemes has substantially expanded in the last years, and studies have focused on health outcomes and outputs [4–8], and some process indicators of quality of care [9] and motivation of health workers. This last aspect is the focus of this paper. Limited but growing evidence exists on the relation between financial incentives and health workers’ motivation and performance, with a focus on whether PBF affects intrinsic motivation [8, 10–14]. The evidence so far tends to look at PBF payments in isolation, without reference to the broader context of the overall remuneration and the other earning opportunities of health workers. However, we know that health workers, and in particular those in low and middle income settings, earn their revenues from a variety of official and unofficial sources, both related to their activities in the health sector as well as those outside [15–17].

In this paper, we aim to fill this gap in the literature by exploring the role of performance-based payments in the context of the entire complex remuneration of health workers, and in relation to the other revenues that they receive [17]. Taking the case of Sierra Leone, where a PBF scheme was introduced for primary healthcare facilities in 2011, this study investigates the absolute and relative contribution of PBF to the overall income of health workers, explores the views of health workers on performance payments, and analyzes their perceptions of revenues and livelihoods with regards to PBF but also in interaction with other incomes. The analysis is based on a mixed-methods research, including a cross-sectional and longitudinal survey and in-depth interviews with public primary health workers, conducted in three districts in Sierra Leone.

### Study setting

In Sierra Leone, following the launch of the Free Health Care Initiative (FHCI) in 2010 which introduced fee exemptions for pregnant women and children under five, a series of complementary reforms has been introduced, many of which addressed issues related to the payment and motivation of health workers [18]. First, in 2010, the public payroll was cleaned to eliminate ‘ghost’ workers and add ‘volunteers’. At the same time, the salary was substantially increased for all technical health workers employed by the Ministry of Health and Sanitation (MoHS). In 2011, a PBF scheme was introduced in primary healthcare facilities nationwide. This scheme was meant to complement the salary increase for staff and substitute the ‘cash to facility’ program in place before [18], and therefore includes a bonus to be shared between facility and staff. Finally, in 2012, a remote allowance for health workers employed in rural regions was also introduced, although it was discontinued towards the end of the same year, because of cash-flow issues. These reforms profoundly reshaped the remuneration of health workers, into a rational and coherent (at least in the design) package [19]. To align to the governmental policies on incentives, donors and many NGOs also gradually eliminated most of the top-ups payed to health workers, and often to those in charge of disease-specific services (e.g., TB and HIV/AIDS).

The PBF design was purposefully simple and the scheme was introduced nationally without piloting, but limited to primary healthcare facilities and not including hospitals (later, a pilot scheme was also created for two hospitals in Freetown). Primary facilities include three types of health centers: Community Health Centers, Community Health Posts and Maternal and Child Health Posts^1^. The performance bonus is calculated quarterly as a fee-for-service payment based on the number of services produced for six indicators (Table 1) [20]. The bonus accrued from the quantity of services provided is then multiplied by a quality score, calculated based on a pre-defined checklist. The checklist includes items concerning the proper completion of the relevant registries for family planning, antenatal and postnatal visits, immunization, under-five consultations, the correct use of partograph for each delivery, the existence of a suitable environment for delivery (cleanliness and availability of equipment) and of a cold chain for immunization. The verification of quantity (by cross-checking the facility registry and declaration) and quality (by compiling the checklist) is performed by the District Health Management Team (DHMTs) in collaboration with the Local Council, the administrative body at district level. The performance bonus is paid into the facility’s bank account every quarter, and can be used for two purposes: a minimum of 40 % of the bonus has to be used to cover the facility’s running costs and small investments (e.g., sanitation and hygiene materials, furniture and small equipment, transport and communication means, stationery, repairs, etc.) and for the payment of casual staff, such as traditional birth assistants (TBAs) and community health workers (CHWs). A maximum of 60 % of the bonus can be used to pay performance bonuses to staff. The bonus for each health worker is determined according to a ‘points’ system based on the cadre of the health worker. For example, Community Health Officers (CHOs) and midwives receive 10 points, Community Health Assistants (CHAs) and nurses 9 points and Maternal and Child Health (MCH) Aides 8 points. Nurses in-charge of the facility, whatever their cadre, receive 2 extra points [20].

**Table 1:** Indicators included in the PBF scheme

Indicator	Payment per service provided	
	Leones	USD
New and current users of family planning	1,000	0.25
Pregnant women completing four antenatal consultations	6,000	1.40
Women in labor assisted by skilled personnel at facility	10,000	2.30
Women completing three postnatal consultations	6,000	1.40
Children under 12 months completing their immunization course	6,000	1.40
Outpatient visits of children under 5 years	300	0.07

Note: Exchange rate at the time of data collection (October 2013): 1 USD = 4,270 Leones

Despite its simple design, the implementation of the PBF scheme faced numerous challenges. An external evaluation by an international NGO was performed in April 2014 looking over the 2 years of scheme implementation. It revealed the weakness of the verification process and found large discrepancies between the indicators verified internally by DHMTs and Local Councils and used to calculate the performance bonuses. Moreover, practical and logistic challenges in the verification procedures resulted in delays of about 1 year in the payment of the performance bonus [21]. At district level, there is evidence [22] that the implementation of the PBF scheme depended on the presence of NGOs operating there. In particular, in Kenema, an NGO was supporting the PBF scheme, by contributing the logistic and financial means that the DHMT needed to carry out verification and supervisions, as well as by providing facilities with training, equipment and drugs focused specifically on the services included in the PBF scheme. In the districts of Bo and Moyamba, NGO support to facilities was more fragmented and not focused on PBF and PBF indicators.

## Methods

This study is based on a mixed-methods research carried out in the districts of Bo, Kenema and Moyamba, between September 2013 and April 2014. Quantitative data were collected from about 200 primary healthcare facilities, where 266 health workers were interviewed. The sample includes the cadres of trained nurses working in health centers, i.e. CHOs (*n* = 30); CHAs, nurses and midwives (grouped together in the analysis) (*n* = 76); and MCH Aides (*n* = 160).

Quantitative data collection consisted in a cross-sectional survey and an 8-week longitudinal logbook collecting data on HWs incomes. Specifically, the survey focused on demographic information as well as on earnings from salary, remote allowance, PBF bonus, share of user fees, top-ups/salary supplementations, per diems, and income-generating activities from outside of the health sector. The longitudinal logbook was left with the health workers to be filled in daily with the activities carried out and all the revenues earned each day. After a preliminary analysis, it was decided to estimate the total monthly income of health workers by using data from the survey where available (i.e. for data on salary, remote allowance, PBF bonus, user fees, salary supplementations, per diems, and non-health incomes), and from the logbooks for earnings from sale of drugs, gifts and payments from patients, and private practice [23]. Based on these data, we calculated the average monthly amount for each income for each individual health worker, including for PBF bonuses. We then computed their importance relative to total income, and estimated two series of regressions, using both standard models and multi-level ones to account for regional differences. We applied a logistic model to explore the determinants of the likelihood of receiving a PBF bonus, and we ran a linear regression of the amount of PBF bonus received to explore the relative importance of individual and facility-level factors. In the case of the linear regression, the multi-level model with fixed effects at district level proved superior to the traditional regression (*p* < 0.0001), and we report results from that model. At individual level explanatory variables include gender, age, cadre, and role within facility, while at facility level they include type and size of facility, urban/rural, and district. Data on other factors which could influence the amount of PBF bonus via the quality component, such as correctly filled-in registries and availability of essential drugs, equipment and infrastructure were not collected.

Qualitative data were collected based on two rounds of semi-structured in-depth interviews with health workers. In total, 39 interviews were carried out in November-December 2013 and March-April 2014. Interviewees were purposefully chosen as a sub-sample from the quantitative survey sample reflecting the mix of health workers in the districts, in terms of cadre, rural/urban post, gender, type of facility and district of posting. A semi-structured interview guide was prepared and flexibly used to inform the interviews, while allowing space for new themes and views to emerge. The interviews did not focus exclusively on the health worker views of PBF and the changes it brought, but were more broadly centered on all the different incomes and sources which make up the total remuneration of health workers. Although the topic guide was iteratively adapted during the interviews, the main themes of focus remained (i) the health workers’ income sources (including PBF), and views on level and fairness, and (ii) non-financial features of the incomes which affect the way health workers perceive and use their remunerations. Interviews were then recorded, transcribed and manually analyzed using content framework analysis [24]. Coding was carried out using a series of pre-defined themes (such as, income features (e.g., how it is paid, timeliness, regularity, etc.), health worker income maximization strategies and income uses), as well as by identifying emerging themes in the health workers in their narratives. Critically, ‘motivation’ was one of such emerging themes, in particular with reference to PBF payments. While all incomes are analyzed together in another publication [25], because of the relevance of issues concerning PBF and motivation in the current international debate, a separate analysis is carried out in this paper, specifically referring to the health worker views on PBF.

## Results

### What PBF contributes to overall health worker income

**Fig. 1:**
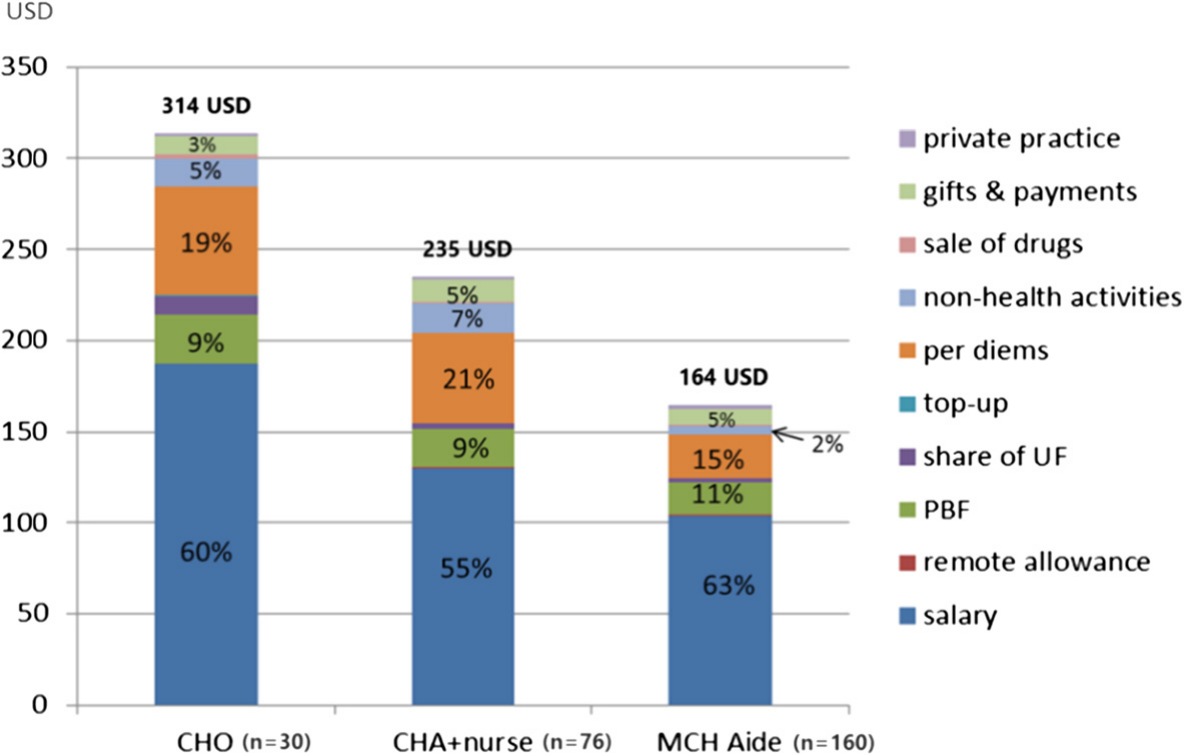
Absolute and relative average income by cadre and by component, including PBF payments (*n* = 266)

The descriptive analysis of the health workers’ overall revenues reveals that PBF accounts for 9–11 % of the total monthly income across the different cadres (Fig. 1). In comparison, salaries are the main source of revenue for health workers, representing between 55 and 63 % of the income, while per diem payments are the second most important and account for up to 20 % of the income. In absolute terms, looking for example at CHAs and nurses, this means that salary accounts for about 130 USD monthly, while PBF bonuses contributes 20 USD per month and per diems about 50 USD (total monthly income is 235 USD). Other revenues, such as those from non-health activities (usually farming or small trading businesses) and gifts from patients (usually in-kind support from the community) are quite important and together add up to about the same amount as PBF payments.

### Determinants of PBF income

The multivariate models presented in Table 2 explore which health workers are more likely to receive PBF bonuses and higher bonuses. Results show that individual factors do not influence these issues, with the exception of being in-charge of the facility. Health workers in charge are more likely to receive a PBF bonus and to receive a higher PBF amount than others. The second finding suggests that the scheme is implemented as designed, since being in-charge grants more ‘points’ in the calculation of their bonus.

**Table 2:** Determinants of receiving a PBF bonus and of the amount received

	(1)	(2)
	Did receive PBF bonus (1 = yes)	Amount of PBF bonus
*Health Worker characteristics*		
Male	0.456 (0.457)	0.253 (0.169)
Age	−0.341 (0.307)	-
In-charge	1.342 (0.386)***	0.343 (0.154)**
*Cadre (omitted category : MCH Aide)*		
Community Health Officers	−0.884 (0.701)	0.105 (0.277)
Community Health Assistants + nurses	−1.057 (0.442)**	0.052 (0.181)
*Facility characteristics*		
*Type of facility (omitted category: Maternal and Child Health Post)*		
Community Health Centre	0.735 (0.506)	0.026 (0.209)
Community Health Post	0.920 (0.415)**	−0.122 (0.158)
Urban	0.189 (0.358)	0.101 (0.138)
*District (omitted category: Moyamba)*		
Bo	−0.199 (0.328)	
Kenema	0.677 (0.351)*	
Constant	−0.609 (0.457)	11.293 (0.219)***
Obs	266	163
Log-likelihood	−163.335	-
Proportion of correct answers predicted	65.8 %	

Note: Standard errors in parenthesis. ***p < 0.01, **p < 0.05, *p < 0.1

Model (1) is a normal logistic regression as the multi-level model with regional fixed effects was not statistically superior, Model (2) is a multi-level linear regression with district fixed effects, which is statistically superior to a standard mode

Looking at facility-level characteristics, it emerges that health workers posted in Community Health Posts are more likely to receive a PBF bonus, thus pointing to unexpected differences between facilities in the implementation of the scheme. Finally, in the logistic model there are important variations at district level, as health workers are more likely to receive PBF bonuses in Kenema. These patterns across district confirm what was found at meso-level about the implementation of PBF and the effects of the presence of NGOs supporting the scheme [22]. In particular, in Kenema, it is possible that the presence of one NGO providing support to all primary facilities, as well as to the DHMT, improved the implementation of the verification procedures and of the payments, and therefore health workers are found to be more likely to receive PBF bonuses.

### Health worker views of PBF and motivation

While the quantitative data allow us a preliminary understanding of the relative importance and the potential motivational impact of PBF, the qualitative interviews complete and enrich this picture. Overall, health workers had positive views on the idea of being paid based on their performance, and reported that effort exerted increased following the introduction of performance-based payments. Health workers said:

“It [PBF] helps us improve more in our work. […] You will put more effort” (CHA/nurse in Kenema)

“We work harder [with PBF]” (CHA/nurse in Kenema)

“It’s a good system because then you perform. If you do not perform well, you do not receive the money” (MCH Aide in Kenema)

“PBF motivates us. Where do I feel there is a lack? Why are my friends getting more than me? What was my problem? Then you sit down and check yourself” (MCH Aide in Kenema).

It is interesting to note that all those quotes were from health workers posted in Kenema, where measures to complement the PBF scheme were supported by an external NGO covering all facilities. Moreover, the last quote points to the fact that PBF entail a self-reflective process in which in-charges compare themselves to their colleagues in other facilities to improve service delivery.

When asked about their views on PBF as an income, health workers seemed to be satisfied and positive, especially if compared to the generally negative comments on their salary, even if the latter is their main source of income. Health workers said that PBF “helps” (two MCH Aides and a CHA/nurse in Bo, Kenema and Moyamba), is “good money” (two CHA/nurses in Bo and Kenema), or “really enough” (MCH Aide in Kenema). The positive perception of PBF compared to salary seems to be linked to the fact that PBF bonuses, given the unpredictability of the payment, are seen as complement or windfall, with less sense of entitlement compared to the salary. A health worker said,

“It [PBF] is manageable, it is just an addition” (CHA/nurse in Kenema) [where ‘manageable’ indicates that its level is financially satisfying]

Despite these positive views, when directly probed on whether they would prefer a salary increase or an increase in PBF fees allocated for each service provided, health workers agreed that they would prefer a salary increase:

“I would prefer an increase in salary, because PBF depends on us, on the way we work” (MCH Aide in Kenema)

“We have PBF but not all the times. Instead of waiting for PBF, let our salary increase” (MCH Aide in Kenema)

### Non-financial aspects of PBF found to motivate health workers

During the interviews, health workers spontaneously mentioned that non-financial aspects of the PBF scheme contributed to their increased motivation and performance. In the quotes below, they reported that they understood better their tasks and responsibilities, because of how the service delivery requirements are detailed in the PBF contracts and in the quality checklist.

“PBF is good, but not only the money. You receive the money and you eat it, but when you are used to [fill in] the partograph, then you enjoy your job” (MCH Aide in Moyamba)

“I prefer PBF because it helps me. Now I know what to do and what not to do” (MCH Aide in Kenema)

Health workers also found motivation through the improvements in the working environment that are paid for with the facility component of the bonus. One health worker said,

“The part used for the facility is motivating. We are improving, we are managing the center” (CHO in Bo).

### Other features of PBF payments that (de)motivate health workers

Moreover, PBF bonuses also have features of which health workers can take advantage within their income utilization strategies. As the quarterly PBF bonus can be a relatively substantial amount of money earned at once, compared to other incomes which are paid in a more fragmented way, it can be saved from family pressures and routine expenditures, and reinvested in non-health income generating activities. An analysis of the quantitative survey data confirmed that income from non-health activities were significantly higher for those with higher PBF bonuses (*p* = 0.05). In turn, revenues from non-health activities provide a certain income stability which allows dealing with the instability of most incomes (e.g. PBF and per diems). One health worker explained,

“I do some little trading, I plan to buy palm oil [with unspent PBF money] and store until the price is favorable. I don’t really schedule because PBF doesn’t come every day, but whenever money is coming you get uses” (CHA/nurse in Bo).

On the other hand, a complaint concerned how PBF is shared -or not- by in-charges with the other workers. In other cases, though, sharing practices, highlighting the existence of team spirit within facilities, were found, in particular in health centers with fewer staff. In some cases, health workers posted immediately after training were given individual bonuses despite not being eligible for it as not working in facility when the bonus was accrued. This practice was justified by the fact that they were not yet on payroll and would have little alternative financial means to support themselves.

“Last time I went for a meeting, there was a lady who went with a complain, saying that her colleague did not recognize her with the PBF” (MCH Aide in Bo).

“She [the in-charge] is encouraging me by giving it [share of PBF] to me” (MCH Aide in Bo).

“She [the other nurse] was not here, but even if when I receive PBF I give her something” (MCH Aide in Moyamba).

### PBF implementation issues and HW motivation

One of the problems indicated by health workers are the long delays in the payment of PBF. Those delays are a key issue that affects the scheme, because they effectively remove the link between effort at facility level and payment provided. Moreover, the delays in payment entail complicated bonus sharing practices. Indeed, often those who worked in the facility when the bonus was accrued are posted elsewhere by the time of its receipt after 1 year. As a consequence, ‘old’ and ‘new’ staff in the facility have to travel and meet in person, in order to make sure the payment is shared with the worker entitled to receive it. This system is extremely complicated given the absence of bank transfers and the difficulties in communication and travel in Sierra Leone, and it relies on the transparency of the in-charges in informing and tracing the staff to provide them the correct payment. Instances of misappropriation or mismanagement of PBF bonuses by some in-charges, as well as implementation failures and mistakes have been recounted during the in-depth interviews:

“I have no access [to PBF] now, because the nurse that was in this center before took the registry and went with it” (MCH Aide in Moyamba).

“12 health centers where left out [never received PBF payments – out of 99 in Moyamba district]. Maybe the computer jumped our name…? We don’t know” (MCH Aide in Moyamba).

Another issue around PBF which limit its potential for motivating health workers are the difficulties they experience in accessing the payment. The bonus is received via the facility bank account which is usually located in the district town, far from the rural facilities, and there is often no information on when it will be paid. In the quote below, a health worker in Kenema recounts the problems in accessing PBF:

“PBF does help actually, but the time to get out PBF is our problem. Because the time when it [the PBF bonus] comes, we have to go through a lot of process before ever accessing it. Certain times you pay transport to Kenema and be there for 1 or 2 days and you are not able to access the money, or they tell you to come another time” (CHA/nurse in Kenema).

## Discussion

The findings presented in this paper show the potential for motivation of performance-based pay in Sierra Leone and of the possible paths through which PBF can motivate health workers, as seen from their own perspective. The analysis allows us a first understanding of the relative importance of PBF, in comparison with the other, formal and informal, income sources. It emerges that, in the context of primary healthcare facilities in Sierra Leone, PBF payments are of a relatively small amount (about 10 %) compared to the overall income. However, as found in other settings [26], other factors beyond the monetary face value influence the perceptions of health workers. PBF as a scheme seems to be well perceived and relatively motivating for health workers because of some non-financial features in its design. One of these is the clarification of responsibilities and tasks in service delivery, which resonates with similar findings in the context of Burundi [27]. Another is the perceived improvement in the physical working environment thanks to the facility component of the bonus, which is also noted in Nigeria [26] and Malawi [28]. Our findings highlight a tension in the narratives of the health workers between seeing PBF bonuses as a reward for effort and viewing them as a windfall, as both notions are there for the staff. Often, the unexpected addition to the income provided by PBF payments is seen as a windfall, which takes a different place in the “mental accounts” of health workers and can be spent differently [25]. Health workers also take advantage of the fact that the amount of the payment can be substantial within their income utilization strategies, where PBF is useful to complement and balance the features of other incomes. The strategies for differential use of different incomes by health workers in Sierra Leone are further explored in another publication [25].

On the other hand, a series of design and implementation issues act as ‘demotivators’ and limit the motivational effects of the incentive. Delays in the payment of the PBF bonus, due to lengthy verification procedures or other issues, are reported across different schemes [26, 29–31] and acknowledged to be a major challenge, in particular because of the disconnection that they cause between effort/performance and payment which is in fact a key point in the theory of change of PBF incentives. Another tension in PBF schemes comes from the fact that performance is measured at facility rather than individual level, and that individual rewards are calculated afterwards, either based on cadre and hierarchy as in the case of Sierra Leone, or on a measure of individual effort, as done in other contexts [26, 30]. A previous study in Sierra Leone [32] found that health staff were motivated by PBF but frustrated by the erratic and unpredictable nature of the payments and because the bonus is shared based on cadre, systematically privileging those in-charge. As found in another setting [30], our findings show mixed results regarding the potential of performance bonuses paid to the facility for the motivation or demotivation of the staff as a team. Some health workers reported sharing payments with those newly arrived who are not entitled to them, pointing to collaboration and reciprocity between staff, while others stressed their discontent for not receiving their rightful bonus. MoHS staff at central level mentioned that “PBF is seen as motivating, but not fair”^2^. While it has been explored in high-income settings [33] and in experimental economic studies outside of the health sector [34, 35], the impact on motivation and performance of the sharing practices within the facility team is an issue which deserves further research across PBF schemes and in low-income settings [14]. Finally, compared to other countries [26, 36], health workers in Sierra Leone did not raise the issue of being demotivated by increased workload linked to PBF, likely because PBF was introduced relatively shortly after the introduction of the free health care initiative which had already entailed a substantial increase in patient load [19] and possibly also because PBF as implemented in Sierra Leone did not create additional reporting requirements.

Our study has some limitations. Despite the use of different techniques (i.e., survey, longitudinal logbook and in-depth interviews) to triangulate information and avoid biases, one issue concern the reliability of data on income amounts, especially for the most sensitive ones (user fees, gifts and payments from patients, sale of drugs, etc.). However, we consider that for official incomes, such as PBF, the estimate is likely to be reliable. In terms of data analysis, variables at facility level concerning the availability of filled-in registries, drugs, equipment, infrastructure, and other factors which could influence the amount received as PBF bonus were not included. However, we provide qualitative information in terms of the varying support that facilities receive from NGOs in the districts which could partially explain some of the differences. Finally, the results discussed above are closely related to the specific context, as well as the design features and implementation challenges of the PBF scheme in Sierra Leone and may not be valid for other contexts and PBF schemes. This stresses the importance of understanding the setting and the specific challenges in the study of PBF schemes.

Beyond the context-specific findings, what our study points to in a generalizable manner is the importance of research focusing not only on the outputs and outcomes of a PBF scheme, but also on the design and the implementation details [30, 31], in order to unpack and understand the underlying mechanisms by which PBF can motivate or demotivate health workers in practice, and from their own perspective. Our analysis also stresses that the remuneration of health workers is complex and interrelated, so that not only the monetary value of the financial incentive is relevant for their motivation, but also other features of the payments, which affect the way they are utilized and perceived by the health workers.

## Conclusions

This study provides a description of the absolute and relative importance of PBF payments within the income of public primary healthcare workers in Sierra Leone and of their views on the motivation provided by performance payments in the context of the overall revenues and incomes.

For policy makers in Sierra Leone, these findings are particularly relevant for the current post-Ebola Virus Disease (EVD) health system strengthening efforts. During the EVD outbreak, the PBF scheme continued to function, although under an even simpler model (e.g. payments were based on data of the health information system with no verification performed) [37], which is likely to have heightened the problems observed above and created others. At the same time, a new scheme was briefly piloted by an NGO in 2015 in the district of Bombali under a different design (so-called “PBF Plus”). While this pilot addressed some of the issues of the ‘simple’ PBF scheme, other challenges emerged, in particular concerning costs and sustainability. At the moment, the future of PBF in Sierra Leone is uncertain and potential new models are being discussed. Given the critical role that PBF seem to play for health workers and facilities despite the numerous weaknesses, it is important that the future development of the PBF scheme capitalize on the lessons learned and builds on them to guarantee an effective role of PBF towards health system strengthening.

Overall, the results confirm the importance of looking beyond each single financial incentive available to health workers separately, but to include all incomes and explore the interrelated dynamics between them which contribute to motivation and performance at individual and team level. As health workers put in place compensating and coping strategies for income use, it is important that researchers and policy-makers look at the effects on motivation of each revenue stream (including performance payments) in relation to one another and considering the broader incentive environment. Findings also stress that the implementation of a PBF scheme at national, district and facility level has a critical impact on the ways it motivates or demotivates health workers and, therefore, must be thoroughly researched in order to assess the impact of PBF.

## Additional files

Additional file 1: Questionnaire and weekly logbook. (PDF 368 kb) Additional file 2: Dataset. (CSV 105 kb)

## Footnotes

1 Maternal and Child Health Posts are the lowest level of primary healthcare centers in the health system, and provide only services related to immunization and child health, delivery and maternal health, family planning and nutrition. They are staffed by one or two nursing aides (Maternal and Child Health Aides). At a higher level are Community Health Posts and Community Health Centres provide a broader range of curative services and are staffed with non-physician clinicians (Community Health Officers/CHOs and Community Health Assistants/CHAs), nurses and midwives. Community Health Centres are the largest health centers of all and usually headed by CHOs, who are also responsible for supervising the Maternal and Child Health Posts in their area.

2 Personal communication with MoHS
