# Supplementary material for: Performance-based financing in the context of the complex remuneration of health workers: findings from a mixed-method study in rural Sierra Leone
Source: BMC Health Serv Res. 2016 Jul 19;16:286. doi: 10.1186/s12913-016-1546-8 (PMC4952280; doi:10.1186/s12913-016-1546-8)
Supplement: Additional file 1: — Questionnaire and weekly logbook. (PDF 368 kb) [file 12913_2016_1546_MOESM1_ESM.pdf]

INITIAL QUESTIONNAIRE – COHORT STUDY

Respondent code:<br>[__ __ __ __]<br>*(District code + enumerator code + HW number according to sequence of interviewing)*	
District:	
Type and name of facility:	
Date of interview:	

1. RESPONDENT DETAILS:

I would like to start by asking some general questions about you:

1.1	Mark respondents' sex	01 [___] Male 02 [___] Female
1.2	What is your marital status?<br>01 [___] Single 02 [___] Co-habiting 03 [___] Married<br>04 [___] Divorced 05 [___] Widowed	
1.3	How old are you?	[___ ___]<br>*99 = Don't Know*
1.4	What is your district of origin?	[_______________________________________]
1.5	What is the last grade of formal education you completed?<br>01 [___] MCH Aide certificate 02 [___] Certificate in Nursing<br>03 [___] Diploma in Nursing 04 [___] Degree in Nursing<br>05 [___] Certificate in Midwifery (SRN) 06 [___] Certificate in Midwifery (SECHN)<br>07 [___] CHO Diploma 08 [___] CHA Certificate<br>09 [___] Other. Specify: [___________________________________]	
1.6	What type of facility do you work in?<br>01 [___] CHC 02 [___] CHP 03 [___] MCHP<br>04 [___] Other. Specify: [_______________________________________]	
1.7	What is your professional title?<br>01 [___] CHO 02 [___] CHA<br>03 [___] Nurse (RN) 04 [___] Midwife (RN)<br>05 [___] Nurse (SECHN) 06 [___] Midwife (SECHN)<br>07 [___] MCH Aide/Nurse 08 [___] Other (specify:)<br>[_______________________________________]	
1.8	What is your Grade?<br>[*should be between 2 and 5*] [___] [*don't know = 99*]	
1.9	What is your post or title within the facility?<br>01 [___] In-charge 02 [___] Staff member<br>03 [___] Other. Specify: [____]	

1.10	Do you have a specific role or duty within the facility? For example, you focus on specific services or wards?<br>01 [__] No, I work on all services/wards every day → *go to question 11*<br>02 [__] I rotate between services and wards → *go to question 11*<br>03 [__] Yes, I work ONLY on some specific services / wards → *go to question 10b*
1.10b	If you work only on SOME specific services / wards, please list them: [*tick **all** relevant*]<br>[__] HIV/AIDS<br>[__] Malaria<br>[__] TB<br>[__] Family Planning<br>[__] ANC / PoNC (antenatal care and post-natal care – pregnant and lactating women)<br>[__] IMCI (integrated management of child illnesses – children)<br>[__] EPI (vaccines)<br>[__] Nutrition<br>[__] Other. Please, specify: [____]
1.11	How many professional health staff work in this facility (ie. do not count the guards/helpers/cleaners)?<br>[*fill in the number*] [__ __ __]

2. INCOME COMPONENTS

		**When?** (mm / yyyy)	**Amount received (Le.)**	**For which period?**
2.1	**Salary** *(last received)*			
2.2	**Remote Area Allowance** *(last received)*			01 [___] One off<br>02 [___] Previous week<br>03 [___] Previous month<br>04 [___] Previous quarter<br>05 [___] Other (specify):
2.3	**Performance-based Financing (PBF)** *[individual bonus!]* *(last received)*			01 [___] One off<br>02 [___] Previous week<br>03 [___] Previous month<br>04 [___] Previous quarter<br>05 [___] Other (specify):
2.4	**Payment from facility revenues distributed to staff** *(last received)*			01 [___] One off<br>02 [___] Previous week<br>03 [___] Previous month<br>04 [___] Previous quarter<br>05 [___] Other (specify):
2.5	**Top-up / salary supplementations** *(last received)*			01 [___] One off<br>02 [___] Previous week<br>03 [___] Previous month<br>04 [___] Previous quarter<br>05 [___] Other (specify):

		Amount received (last month) - Le.
2.6	**DSA for training, workshops, etc.** *(received last month) [entire amount received]*	
2.7	**Income-generating activities <u>outside</u> the health sector** *(received last month)* [*for ex, trading or selling business, farming, etc.]*	

→ Thank the respondent. Now introduce and explain the logbook.

WEEKLY LOGBOOK - COHORT STUDY

Respondent code:<br><br>*(District code + enumerator code + HW number according to sequence of interviewing)*
District:
Type and name of facility:
Date when logbook was left with the respondent:

Thank you for your cooperation.

We are interested in better understanding your **work management** (i.e., the way you divide your time between activities) and **workload** throughout the day.
Don't forget this is an **anonymous** questionnaire and all information you provide will be kept **confidential** – for more information on this ask the enumerator (contact below).

INSTRUCTIONS:

1. Begin filling in this logbook ***today*** and continue to record your activities and income until the end of the week.
2. Fill in the table daily, **at the end of each day of work**.
3. Once finished a booklet, use a new one. Use **one logbook per week** and record activities and income over the next ***9 weeks***.
4. For each time period you worked on an activity, you should fill in the logbook detailing the **activity,** the time spent on it and the amount earned (if any). **The list of activities is below**. Use it as a reference when filling in the logbook.

   ---------------------------- ***There is an example overleaf to guide you*** --------------------------------------

5. If you have any queries or doubt, do not hesitate to call us or send a text (you will be called back) to :

   [________________________________]   *Add name and phone num of enumerator*

LIST of TYPES OF ACTIVITIES

Activities WITHIN the health facility

- General (integrated) clinical activities (covering different problems of a patient. For ex: outpatient visits, visits of children or pregnant women)
- Disease/service/program specific activities (for ex: Family Planning, TB, HIV/AIDS, etc) → *Please, specify in the logbook which disease/service/program.*
- General administrative work (for example HIS reports, pharmacy records, etc.)
- Disease/service/program specific administrative work (for example, HIV or other disease specific information reports). *Please, specify which disease/service/program.*
- Meetings within the facility (for ex: meeting with the staff to agree on shifts, etc.)
- Night guards

Activities OUTSIDE the health facility

- Outreach activities in the community
- Meeting outside of the facility. *Please, specify type/topic of meeting, organiser and meeting place*
- Training, workshop, etc. *Please, specify topic of workshop, organiser and place*
- Private practice (health-related work that you do in a clinic, at home or at the home of a patient)
- Other non-medical activities that generate income (for ex: trading, business, farming). *Please, specify***.**

EXAMPLE **Daily activity logbook – WEEK # 1** **EXAMPLE**

Fill in the date on which you started recording: Monday, September 16th

TIME	Monday	Amount earned	Tuesday	Amount earned
7am				
8am	Meeting in facility:			
9am	planning of week			
10am				
11am	Outpatient consultations	Gifts: 20,000 Le.		
12pm	Consultations: Family		Training:	DSA: 50,000 Le.
1pm	Planning		- Family Planning	
2pm			- Funded by UNFPA	
3pm			- at DHMT Office	
4pm	Private practice	30,000 Le.		
5pm				
6pm	Work in shop	10,000 Le.		5,000 Le.
7pm			Work in shop	

Daily activity logbook – WEEK #______

*Date on which you started recording:*___________________________________

TIME	Monday	Amount earned	Tuesday	Amount earned
Early morning				
7am				
8am				
9am				
10am				
11am				
12pm				
1pm				
2pm				
3pm				
4pm				
5pm				
6pm				
7pm				
8pm				
Night				

TIME	Wednesday	Amount earned	Thursday	Amount earned
Early morning				
7am				
8am				
9am				
10am				
11am				
12pm				
1pm				
2pm				
3pm				
4pm				
5pm				
6pm				
7pm				
8pm				
Night				

**TIME**	**Friday**	**Amount earned**	**Saturday**	**Amount earned**	**Sunday**	**Amount earned**
Early morning						
7am						
8am						
9am						
10am						
11am						
12pm						
1pm						
2pm						
3pm						
4pm						
5pm						
6pm						
7pm						
8pm						
Night						
